# Autonomic Dysreflexia in the Peripartum Patient: A Multidisciplinary and Interprofessional Simulation Scenario

**DOI:** 10.7759/cureus.1513

**Published:** 2017-07-25

**Authors:** Purnima M Rao, Adam Garber, Chandrew Rajakumar, Genevieve Rousseau, George Dumitrascu, Glenn D Posner

**Affiliations:** 1 Department of Anesthesiology and Pain Medicine, The Ottawa Hospital / University of Ottawa; 2 Department of Obstetrics & Gynecology, The Ottawa Hospital / University of Ottawa; 3 Department of Obstetrics & Gynecology, University of Calgary; 4 Department of Innovation in Medical Education, University of Ottawa

**Keywords:** postgraduate medical education, simulation scenario, obstetrics and gynecology, anesthesiology, nursing, crisis resource management

## Abstract

This case is one of an eight-case multidisciplinary curriculum designed and implemented at the University of Ottawa by simulation educators with specialty training in obstetrics and gynecology (ob/gyn) and anesthesiology. Consultation with a nurse educator maintained quality and relevance of objectives for nursing participants.

The curriculum was prepared to train ob/gyn and anesthesiology residents and nurses to hone crisis resource management skills and to recognize and manage rare/critical medical events in an obstetrical setting. Obstetricians, anesthesiologists, and nurses often work together in acute, high-stakes situations and this curriculum provides a safe environment to practice team-based management of such emergencies.

Over an eight-year period, this curriculum has been executed in scenario couplets on a four-year cycle to allow ob/gyn and anesthesiology residents exposure to all scenarios during a five-year residency beginning in their second year. Prospective evaluation data has been positive. For example, over 90% of participants rated these simulations to be 5 out of 5 for “Was an effective use of my educational time” and “Will influence/enhance my future practice”.

In this scenario, participants must recognize and manage a parturient with spinal cord injury in active labour who develops autonomic dysreflexia. The fetal heart tracing becomes abnormal and the team must respond with urgent delivery. This scenario requires a mannequin for a pelvic exam and a pregnant abdomen.

This simulation case includes a case template, critical actions checklist, debriefing guide, summary of key medical content, and an evaluation form for learners to provide feedback.

## Introduction

Due to improvements in the acute management and rehabilitation of patients with spinal cord injuries (SCI) as well as advances in reproductive technologies, an increasing number of these women are becoming pregnant. Complications of chronic SCI may be exacerbated by the physiologic changes of pregnancy, labour, and delivery. Autonomic dysreflexia (AD) is a common syndrome that occurs in patients with an injury at or above T6, characterized by acute hypertension, bradycardia, headaches, arrhythmias, and in severe cases respiratory failure, intracranial hemorrhage or hypertensive encephalopathy. Pregnant patients will also present with fetal distress due to uteroplacental vasoconstriction. This life threatening presentation, for both mother and baby, must be immediately recognized and appropriately managed. Most trainees and clinicians will see very few such patients in their career, making diagnosis and management challenging.

This simulation scenario was created in order to allow anesthesiology residents, obstetrics residents and nurses to practice the diagnosis and management of a patient with a SCI presenting with acute AD. Effective coordination and communication between these three services is essential to ensure the safety of both mother and baby [1-2].

This scenario is part of a four-year interdisciplinary curriculum for ob/gyn and anesthesiology residents developed at the University of Ottawa Skills and Simulation Centre (see Appendix E). Obstetricians and anesthesiologists often work together in acute, high stakes situations. This curriculum was designed in order to allow residents in both fields to practice both technical and crisis resource management skills in a safe and risk-free environment. Further, the interdisciplinary aspect of this curriculum allows both fields to learn about each other's knowledge, roles, and priorities during a crisis.

The target audience is ob/gyn residents, anesthesiology residents, and practicing nurses as part of a comprehensive interdisciplinary curriculum of theatre-based simulation. Three to five learners participate in each session, and the duration of training is one hour.

The goals of the scenario are for the team to recognize and appropriately manage autonomic dysreflexia in a labouring patient, to manage a patient with a chronic spinal cord injury presenting with an abnormal fetal heart tracing, and to demonstrate effective crisis resource management skills.

Team objectives

(Adapted from the Ottawa Global Rating Scale [3])

1. Demonstrates leadership skills by remaining calm and in control, making firm decisions, and maintaining a global perspective.

2. Demonstrates problem solving skills by conducting a thorough, but efficient airway, breathing and circulation (ABC) assessment using a concurrent management approach and considering most likely alternatives in crisis.

3. Demonstrates situational awareness skills by avoiding fixation error, reassessing and re-evaluating situations, anticipating likely events.

4. Demonstrates resource utilization skills by using resources to maximal effectiveness, setting clear task priority, asking for help early.

5. Demonstrates communication skills by communicating clearly and concisely, encouraging input and listening to staff feedback, using directed verbal/non-verbal communication.

Ob/gyn resident objectives

1. Demonstrate management of hypertension in a labouring patient with a chronic spinal cord injury.

2. Demonstrate an approach to assessment and management of labour in a patient with a chronic spinal cord injury.

3. Recognize and demonstrate the management of an abnormal fetal heart tracing.

Anesthesiology resident objectives

1. Demonstrate the ability to independently provide anesthesia care for patients with spinal cord injury and autonomic hyperreflexia.

2. Demonstrate knowledge of how to develop and execute a plan for general anesthesia with tracheal intubation based on the physiology and physical changes of pregnancy.

Nursing objectives

1. Demonstrates understanding of the knowledge required to meet the needs of complex clients. (Knowledge)

2. Identifies and recognizes abnormal or unexpected client responses and takes action appropriately. (Knowledge application)

3. Manages multiple nursing interventions simultaneously. (Knowledge application)

4. Demonstrate seeking assistance appropriately and in a timely manner. (Knowledge application)

## Technical report

Case summary

A 26-year-old gravida 1 para 0 patient with a history of T5 spinal cord injury due to a motor vehicle accident two years ago presents at 37 weeks gestational age with a new onset headache, which is a result of autonomic dysreflexia (AD) precipitated by labour. After initial assessment by a triage nurse, an obstetric resident is asked to assess the patient and initiate management. Upon pelvic examination, the patient develops worsening AD and fetal bradycardia. This prompts the ob/gyn resident to prepare for an emergency caesarean section and to call for the ob/gyn "attending" and anesthesiologist. Once in the operating room, the fetal heart rate stabilizes, allowing the team to have a discussion regarding the appropriate management of this patient’s labour and delivery. The fetal heart rate once again decelerates prompting induction of general anesthesia for an emergency caesarean section.

Learner preparation

We advocate for a comprehensive pre-briefing prior to any theatre-based simulation session, as described by Rudolph, et al. [4].

Nurse: You are working in obstetrical triage. Your next patient in triage is a 26-year-old G1P0 patient at 37 weeks who is complaining of a worsening headache as well as vaginal spotting.

Junior ob/gyn resident: You are the senior ob/gyn resident on call in a tertiary hospital. Your staff ob/gyn is also in the hospital.

Senior ob/gyn resident: You are the ob/gyn attending physician on call in a tertiary hospital. You have a senior ob/gyn resident also on call with you.

Anesthesiology resident: You are the attending anesthesiologist on call for ob/gyn in a tertiary hospital.

Setup

Equipment/Environment

This scenario takes place during a call shift in a tertiary care hospital obstetrical triage and operating room.

Two rooms are required (#1 set up as an obstetrical triage, #2 set up as an operating room)

OR

A single simulation room (initially set up as an obstetrical triage with hidden operating room equipment in the periphery that will be uncovered and moved into place once the team “enters” the operating room)

Part 1 – High fidelity mannequin (wig, pregnant abdomen with baby inside) on stretcher. Awake, breathing spontaneously with standard monitors available but not connected, monitor at the head of the bed. Fetal heart rate (FHR) monitor beside the patient. Peripheral intravenous (IV) in situ connected to IV fluid.

Part 2 – Mannequin as above on operating room table. Awake, breathing spontaneously with standard monitors attached, arterial line available. Fetal Doppler available. Peripheral IV in situ connected to IV fluid. Anesthetic equipment and surgical equipment available.

Please refer to Appendix A for a detailed list of all required equipment.

Personnel

Simulation instructor

Simulation technician

Confederate nurse - acting as a second nurse in the scenario, and circulating nurse in the OR (optional headset for prompting)

Confederate partner (optional)

Confederate respiratory therapist or anesthesia assistant (optional)

Learners

Senior anesthesiology resident – attending anesthesiologist

Senior ob/gyn resident – attending obstetrician

Mid level ob/gyn resident – obstetric resident

Obstetric nurse – obstetric nurse, functioning as the triage nurse and the patient's primary nurse

The baseline state of the mannequin is described in Table 1.

**Table 1:** Initial Parameters BPM: beats per minutes; HPI: history of present illness; PMHx: past medical history; HEENT: heads, eyes, ears, nose, throat; GU: genitourinary.

Initial Presentation			
Initial vital signs	Blood Pressure: 170/105; Heart Rate: 70; Respiratory Rate: 14; Saturation: 98% on room air; Fetal Heart Rate: 120-140BPM		
Overall Appearance	The patient is lying flat on her back with a cold cloth on their forehead. Blood pressure monitor, saturation probe, and fetal heart monitor have been applied and the monitor is beside the patient. The patient’s partner is by her side looking anxious. The patient is awake, alert, but anxious.		
Actors and roles in the room at case start	Patient – High fidelity mannequin, being voiced from control room, with pregnant abdomen Partner – Confederate actor (could be played by an extra resident or simulation instructor)		
HPI	When asked, the patient will state that she is 26 years old and 37 weeks pregnant with her first pregnancy. She woke up this morning with a headache that worsened throughout the day. She is also sweating and has had a small amount of vaginal spotting. She has no visual changes, epigastric pain or swelling of her legs. She does not feel fetal movements due to her spinal cord injury. She has not noticed any leakage of fluid or rupture of membranes. Her husband will volunteer that they were seen in the pre anesthetic clinic and were told by the anesthesiologist that they needed an early epidural when she went into labour.		
Past Medical/Surgical History	Medications	Allergies	Family History
OB – Her pregnancy has been uncomplicated. Her previous ultrasounds were normal. She had several urinary tract infections during her first and second trimesters but none recently. PMHx- She was involved in a motor vehicle accident 2 years ago which resulted in a T5 spinal cord injury (will provide level if asked). Because of her paraplegia she has recurrent urinary tract infections due to self- catheterization. She has had episodes of autonomic dysreflexia in the past due to urinary tract infections. She has no history of head trauma or migraines or previous headache. No previous surgery.	Prenatal vitamins	None	None, including no history of problems with anesthetics.
Physical Examination			
General	Awake, alert, anxious. Flushed and diaphoretic.		
HEENT	Normal		
Neck	Normal		
Lungs	Breath sounds equal bilaterally and clear to auscultation		
Cardiovascular	Hypertensive, tachycardic, normal heart sounds, equal pulses bilaterally.		
Abdomen	Pregnant		
Neurological	No movement of lower extremities Hyperreflexic in lower extremities		
Skin	Diaphoretic		
GU	N/A		
Psychiatric	Anxious		

Ideal scenario flow

The nurse enters the room (obstetrical triage) to find a pregnant patient, anxious with a headache. She takes an initial history and applies monitors, including a fetal heart rate monitor and recognizes severe hypertension with no fetal distress. The ob/gyn resident is paged to assess the patient. The ob/gyn resident takes a history, orders initial investigations, and orders labetalol or hydralazine to decrease the patient’s blood pressure. They may ask for the anesthesiologist to be paged. The patient develops worsening autonomic dysreflexia, precipitated by a pelvic exam performed by the resident, with a resulting fetal bradycardia. This prompts the ob/gyn resident to call for an emergency caesarean section. At this point, depending on the resources of the simulation centre, the patient is transferred to another simulation room prepared as an operating room or equipment is brought into the room and the team will be told they are now in an operating room. At this time the ob/gyn and anesthesiologist enter the simulation. The team reassesses the patient and recognizes that the fetal heart rate has stabilized. The anesthesiologist assesses the patient and the ob/gyn receives handover from the ob/gyn resident. The team discusses the optimal management of this patient’s labour and delivery. The fetal heart rate once again decelerates prompting induction of general anesthesia for an emergency caesarean section. The scenario ends after anesthesia is induced. Table 2 provides notes for the instructor based on branching points during the scenario based on participant actions.

**Table 2:** Flow of the Scenario BP: blood pressure; FHR: fetal heart rate; PVCs: premature ventricular contractions; NICU: neonatal intensive care team;

Instructor Notes – Changes and Case Branch Points		
Intervention / Time point	Change in Case	Additional Information
*If the team mistakenly diagnoses the patient with pregnancy induced hypertension or any other hypertensive disorder of pregnancy*		*They will be allowed to continue managing according to their differential diagnosis. Their management will be ineffective and the patient and fetus will continue to deteriorate*
*If the nurse or the ob/gyn resident want to move the patient from triage to an inpatient room*		*They will be told that a room is being prepared for the patient but this will take five to ten minutes*
*Two minutes into the case*		*Patient will volunteer that she is paraplegic if this information has not already been elicited* *Husband will say “We were told at the pre anesthesia clinic that she needs an epidural as soon as she goes into labour”*
*If labetalol ordered or given*	*Decrease in BP to 168/95* *HR 60*	
*If hydralazine ordered or given*	*Decrease in BP to 168/95* *HR unchanged*	
*Five minutes into the case OR immediately following the pelvic exam*	*BP 190/115* *HR 50* *FHR decreases to 60 BPM*	*If not recognized by participants within 30 seconds, patient will ask “Is my baby ok?”*
*Once transferred to the Operating Room*	*BP 200/115* *HR 50* *FHR recovers to 110 *	*If team does not recognize recovery in FHR, patient will ask “Is my baby ok?”* *OR* *Confederate nurse will prompt the team to look at the monitor by saying “the FHR has changed”*
*15 minutes*	*Fetal heart rate drops to 60*	*If not recognized by the team, confederate nurse will prompt the team to look at the monitor by saying “the FHR has changed”*
*If general anesthesia is induced *	*BP 185/105* *HR 55* *Patient loses consciousness *	
*If succinylcholine is used during induction of anesthesia*	*Patient develops PVCs and runs of ventricular tachycardia* *The patient’s blood pressure will remain unchanged. *	
*If the anesthesiologist calls for help*		*Confederate respiratory therapist or anesthesia assistant will enter *
*If the team calls for NICU*		*Confederate nurse will say “NICU is on their way”*
*If the team requests more help*		*Confederate nurse will say “I’ve called the front desk but the other team is in the next case room performing another caesarean section and they cannot leave”*
*If the team requests results of the blood work they sent in triage*		*The confederate nurse will provide them to the team once in the operating room. *

Critical actions

The nurse assesses the patient in obstetrical triage and recognizes uncontrolled hypertension in a pregnant patient.

The nurse calls the ob/gyn resident to assess the patient.

The ob/gyn resident assesses the patient in triage, orders initial investigations (complete blood count (CBC), electrolytes, liver function tests, coagulation tests, urinalysis). They order medications to treat the patient’s blood pressure including labetalol or hydralazine.

The ob/gyn resident and nurse recognize the fetal heart rate deceleration and prepare for an emergency caesarean section, calling both the ob/gyn and anesthesiologist.

The anesthesiologist performs a focused assessment of the patient.

The ob/gyn receives handover from the ob/gyn resident.

In the operating room the team recognizes that the fetal heart rate has stabilized.

The team discusses the management of this patient’s labour and delivery, including the options of epidural analgesia, spinal anesthesia, and general anesthesia.

The team recognizes the fetal heart rate deceleration and prepares for an emergency caesarean section.

The anesthesiologist induces the patient using a general anesthetic, using a technique that is sensitive to an existing spinal cord injury and while being sensitive to the severe hypertension by blunting the laryngoscopy response. 

The nurse assists the anesthesiologist in induction of general anesthesia.

The ob/gyn team prepares to perform a caesarean section.

Anticipated management errors

The following is a list of management errors or difficulties that are commonly encountered when using this simulation case.

Failure to Recognize Autonomic Dysreflexia

Many of our learners do not recognize the presentation of autonomic dysreflexia and instead manage the patient as if she has preeclampsia. We specifically cover this information during the initial part of our debriefing and send the learners a handout after the session.

Failure to Recognize Changes in the Fetal Heart Rate

Some of our learners do not recognize the deceleration of the fetal heart rate in triage or the recovery in the OR. We allow the confederate nurse to prompt the learners to look at the tracing.

Assessment & debriefing guide

Assessment

This scenario was developed as a formative assessment tool with a focus on non-technical skills and crisis resource management. There are multiple suitable assessment methods that can be used. We utilize a performance checklist, as well as a Crisis Resource Management Checklist (Ottawa GRS [3]). Please refer to Appendix B for the performance checklist.

Debriefing

We suggest an interprofessional, multidisciplinary team debriefing guided by an experienced simulation instructor. The goal of the debriefing is to allow learners to actively reflect on their own and the team’s performance, which is an essential step in adult experiential learning. Instructors should strive to create a safe, supportive, and respectful environment where all learners are encouraged to participate. Debriefing should focus on the educational objectives, both technical and non technical. We advocate for the use of the promoting excellence and reflective learning (PEARLS) framework to organize the debrieifing [5]. Where available, we advocate for the use of video review during the debriefing. Be cognizant of timing; the debriefing should take 30-40 minutes. Strategies to debrief common errors can be found in Table 3, and a complete debriefing guide can be found in Appendix C.

**Table 3:** Common Errors and Debriefing Strategies

Error Type	Common Errors Observed	Solutions (Teaching Points)
Technical Skills (Medical Knowledge, Clinical Skills)	Does not include Autonomic Dysreflexia (AD) in the differential diagnosis	Directive feedback or group discussion during the debriefing session. Provide reference article on the management of a parturient with a spinal cord injury.
	Does not know correct antihypertensives or doses	Directive feedback or group discussion during the debriefing session. Provide reference article on the management of autonomic dysreflexia.
	Does not recognize or discuss regional anesthesia as an option in a stable patient with AD	Directive feedback or group discussion during the debriefing session. Provide reference article on the management of a parturient with a spinal cord injury.
	Uses succinylcholine during induction of General Anesthesia	Directive feedback during the debriefing session. Refer to reference article or textbook chapter on the contraindications to succinylcholine.
	Fails to recognize patient’s antepartum anesthesiology consult	Increased awareness of the possibility of this diagnosis through simulation. Ensuring thorough review of patient’s chart. Active listening to patient and her support.
Non Technical Skills (Crisis Resource Management)	Fixation on gestational hypertension/preeclampsia	Discuss three types of fixation errors. Discuss tools for avoiding fixation errors, for example, voicing the differential diagnosis out loud, asking for input from other team members, using a systematic and broad approach to the differential diagnosis.
	Lack of resource utilization and mobilization of extra resources - Does not call for help from Attending Obstetrician, Anesthesia Assistant, NICU, etc	Discuss available resources (will be centre specific).
	Lack of communication between team members	Discuss shared mental model regarding the differential diagnosis and/or management plan. Discuss critical moments for sharing a mental model – for example, before/after transfer to the OR, prior to induction of anesthesia. Discuss closed loop communication.
	Failure to recognize changes in Fetal Heart Tracing	Discuss situation monitoring – for example, scanning of monitors at critical moments, before/after transfer to the OR, before/after any significant procedure, during any acute change in vital signs.

Program evaluation

We emphasize collecting evaluative data from participants after their simulation sessions. Our evaluation tool can be found in Appendix D. The results of our initial program evaluation can be found in Table 4.

**Table 4:** Evaluation Data

	1 (strongly disagree)	2	3	4	5 (strongly agree)
The objectives were made clear				3	13
The scenarios were relevant to my practice					16
The simulation team behaved in an appropriate and believable manner during the scenario				3	13
There was sufficient time allotted for hands-on participation and group interaction					16
The staff met the stated learning objectives				1	15
The staff were knowledgeable and informed					16
The staff provided adequate and appropriate feedback				1	15
The debriefing sessions were logically organized and clarified important issues					16
The knowledge gained from this session will enhance/influence my practice					16
The session helped increase my confidence in treating patients when a crisis occurs				2	14
I would like to attend additional simulation sessions		1		1	14

Of the 26 participants, the following identified these CanMEDS 2014 roles as having been addressed during the session	
Medical Expert	17
Collaborator	17
Professional	14
Health Advocate	12
Communicator	18
Manager	12
Scholar	10

Please take a moment to reflect on your previous experience in both simulation and clinical practice. Do you think that simulation has helped your clinical practice?	
Yes	16
No	0

## Discussion

This scenario, as well as the rest of the ‘Ob/Gyn Anesthesia Nursing Simulation Curriculum’ (see Appendix E) (Table 5), was developed to allow learners to practice managing rare and important clinical scenarios that they may not experience sufficiently during their period of training to achieve competence. Autonomic dysreflexia is a rare presentation; however, misdiagnosis and management leads to significant morbidity and mortality for the mother and fetus. This scenario highlighted an important clinical knowledge gap. Furthermore, it allowed for interdisciplinary practice of crisis resource management skills that can be applied to a multitude of clinical situations. This scenario provided an opportunity for the anesthesiology residents to recognize medical expertise they have, which other members of the team may not. In order to provide safe patient care they had to share their mental model effectively with the ob/gyn and nursing teams. The ob/gyn residents and nurses, used to managing hypertension due to pregnancy induced hypertension, were given the opportunity to practice developing a broad differential diagnosis and avoid fixation errors. This was reflected in the positive feedback from learners regarding the clinical relevance of the scenario and applicability to practice. Several learners stated the usefulness of having to diagnose and manage a rare medical condition.

During the development, piloting, and running of this scenario two main challenges were encountered. First, there was a lack of adequate resources to run this simulation using two separate rooms, “an obstetrical triage” and a separate “operating room”. The decision was made to hold the entire scenario in one simulation room. After the decision had been made by the team to proceed for an operative delivery, equipment, which had been hidden under covers in the periphery of the room, was uncovered and positioned to simulate an operating room. This does not appear to significantly affect realism for the learners in the scenario. The second challenge was that the majority of learners did not recognize the patient was at risk for autonomic dysreflexia and proceeded with routine medical management of an obstetrical patient in triage, including a pelvic exam leading to deterioration of the patient’s condition. We had to be sure to address this medical error during the debriefing in a way that was respectful of the students’ experience and the stress of participating in simulation. Overall, the simulation and debriefing was a positive learning experience for the students. A majority of students felt that feedback was given in an appropriate manner and stated that they would like to attend additional simulation sessions.

There are limitations to this modality of teaching. Simulation requires many resources including equipment, instructor and learner time, and instructor expertise. It is also heavily dependent on learner engagement and their willingness to participate and share their experiences with their colleagues. With the appropriate support from departments and learners along with adequate preparation, we feel that it is an invaluable resource especially for teaching around rare medical conditions and crisis resource management skills.

## Conclusions

We describe the design and implementation of an interprofessional and multidisciplinary simulation scenario to teach ob/gyn residents, anesthesiology residents, and nurses about the management of autonomic dysreflexia in the peripartum patient. Debriefing points and common pitfalls are elaborated along with program evaluation data. This scenario is one component of a comprehensive theatre-based simulation curriculum, the goal of which is to provide formative assessment around less common obstetrical emergencies, and crisis resource management.

## Appendices

Appendix A: Equipment

General

2 simulation rooms (#1 set up as an obstetrical triage, #2 set up as an operating room (OR))

or

1 simulation room (initially set up as an obstetrical triage with hidden OR equipment in the periphery that will be uncovered and moved into place once the team “enters” the OR)

Mannequin

- SimMom (Laerdal Medical Corporation, NY, USA) (wig, pregnant abdomen with baby inside) on stretcher, awake, breathing spontaneously

- 1 x 18G IV in situ connected to 1L crystalloid

- Wheelchair at bedside

- Patient chart with blank triage records

Monitors

Initial:

- NIBP cycling every three minutes

- SaO2

- EKG

- FHR monitor

Operating room:

- NIBP cycling every three minutes

- SaO2

- EKG

- FHR monitor

- Arterial line (available, not attached to mannequin)

Anesthesia Equipment (in OR)

Ventilator and anesthetic machine turned off:

- Standard monitors as above

- Breathing circuit and mask

- Suction and Yaunker

- Gas supply for air, O2

- Anesthetic agent (sevoflurane or desflurane)

Airway:

- Laryngoscope

- Oral airway

- endotracheal tube (ETT) (#6, #7)

- Stylet

- Bougie

- Laryngeal mask airways (LMA) (#4, #5)

- Glidescope

Breathing:

- Stethoscope

Circulation:

- 1L bags of crystalloid x 2

- IV infusion line x 2

- Blood set x 1

Induction drugs:

- Fentanyl or sufentanil

- Propofol

- Ketamine

- Succinylcholine, rocuronium

Antihypertensive medications:

- Labetalol, esmolol

- Nitroglycerine

- Hydralazine

- MgSO4

Various size syringes:

Spinal set up

Epidural set up

Surgical Equipment

- Surgical drapes

- Gowns, gloves, OR masks

- Simulated abdominal antiseptic prep (empty bottle of prep)

- Copy of surgical safety checklist

- Cesarean section tray (NOTE: a basic operating tray should be present to adequately simulate an obstetrical operating room. However, the actual steps of cesarean section are not part of the objectives for this scenario.)

- Lap-pads/Sponges

- #10 Blade on handle

- 2x Kocher clamps

- Doyen’s retractor/lower-end retractor/large abdominal wall retractor

- 4x Kelly clamps

- 1x Umbilical cord clamp

- 2x Green-Armytage clamps

- Needle driver

- Suture (does not need to be opened)

- Wedge

- Laparotomy drape + clips x2 (to hang)

Personal protective equipment

- Gowns x4

- Gloves x4 pairs

- Caps/Bouffant

Appendix B: Performance checklist

Anesthesia

Performs a focused history and physical examination eliciting relevant information regarding:

- Current pregnancy

- Complications relating to chronic spinal cord injury

- Cardiovascular status

- Type of treatment initiated and patient’s response

Initiates resuscitation of mother and fetus:

- Ensures IV access, CAS monitoring, supplemental oxygen and fetal heart rate (FHR) monitoring

- Ensures the patient is appropriately positioned to minimize aortocaval compression

Recognizes autonomic dysreflexia;

Initiates treatment for autonomic dysreflexia:

- Minimizes noxious stimuli exacerbating autonomic dysreflexia by ensuring a functioning Foley catheter, preparing for labour analgesia, communicating with ob/gyn team to minimize pelvic examination

- Provides intravenous medication to lower blood pressure such as hydralazine, nitroglycerine, and nitroprusside

Conducts safe induction of general anesthesia for emergent caesarean section:

- Calls for help

- Prepares for potentially difficult airway with appropriate positioning and ensuring airway adjuncts available

- Ensures entire team is prepared for immediate caesarean section prior to induction of anesthesia

- Avoids succinylcholine due to chronic spinal cord injury

Obstetrics

Performs a focused history and physical examination eliciting relevant information regarding:

- Current pregnancy

- Complications relating to chronic spinal cord injury

Responds to patient’s concerns about requiring early epidural;

Initiates resuscitation of mother and fetus:

- Ensures IV access is established and left lateral tilt is employed to minimize aortocaval compression

- Ensures continuous fetal monitoring

- Initiates management of hypertension

- Requests assistance in medical management of complex patient

- Recognizes abnormal fetal heart tracing and declares need for urgent delivery by cesarean section

- Collaborates with anesthesia and nursing to manage autonomic dysreflexia in the context of urgent cesarean section

Nurse

Performs an efficient assessment of a triage patient;

Seeks help early in the assessment of this patient;

Assists with initial intrauterine resuscitation;

Collaborates with obstetrics and anesthesia during urgent cesarean section.

Appendix C: Debriefing guide

The following is adapted from the PEARLS Debriefing Guide [5]

Pre Briefing

Prior to the session, the instructor should conduct an orientation session covering the following:

Confidentiality: instructors should sign a confidentiality form agreeing not to discuss the learners’ performance during the scenario or discussion during the debriefing outside of the simulation environment. Learners should sign a confidentiality form agreeing not to discuss any aspects of the case or debriefing outside of the simulation environment.

Equipment: instructors should describe the function of the simulation mannequin (what types of procedures can be performed, where to feel for pulses, where to listen for breath sounds, etc), how to operate the monitors in the room, how to get extra resources (location of phone, phone number to call, resuscitation cart, difficult airway cart, etc).

Fidelity: the learners should be encouraged to treat the scenario as they would a real clinical experience. Learners should be advised that if they are unsure that a clinical finding or event is intended or an artifact, to state this aloud so they can be appropriately directed.

Debriefing

Reactions phase: allows learners to express their initial thoughts and feelings

Tips:

- Ensure all learners are able to express themselves

- Make note of any positive or negative emotions, major conflicts or tensions that can serve as a point of discussion during the ‘Analysis’ phase

- Ask open-ended questions: “How are you feeling?”

Descriptive phase: ensures all participants understand the major medical issues and key events of the case

Tips:

- Ask one or two participants to summarize the case in two to three sentences to avoid a detailed play-by-play of the scenario

- Ensure participants from all disciplines are able to contribute

- Make note of any disagreements between the learners as to the medical content (differential diagnosis, management plan, etc) which can serve as a point of discussion during the ‘Analysis’ phase

- Clarify any misconceptions, correct any errors regarding the main presentation and diagnosis, i.e.“This case was meant to portray a patient with a chronic spinal cord injury presenting with severe autonomic dysreflexia due to labour”

Key Clinical Points

Anesthesiology:

Autonomic dysreflexia may occur in patients with spinal cord injury above T5 and is due to a loss of control of sympathetic spinal reflexes distal to the spinal cord injury. Noxious stimuli below the level of injury causes reflex sympathetic activation, catecholamine release and vasoconstriction resulting in hypertension, arrhythmias, flushing, respiratory distress, and in the pregnant patient, uteroplacental vasoconstriction and fetal hypoxia.

Any noxious stimuli should be avoided or immediately treated, including the stimulation caused by uterine contraction.

Spinal or epidural anesthesia will prevent autonomic dysreflexia by inhibiting the stimulation caused by uterine contraction.

Autonomic dysreflexia should be treated with titratable agents such as nitroglycerin, hydralazine, and nitroprusside.

Obstetrics:

Labour or cervical examination can stimulate or worsen autonomic dysreflexia in patients with longstanding spinal cord injuries.

Early regional anesthesia (spinal or epidural) is the preferred approach to effectively treat autonomic dysreflexia and maximize opportunity for spontaneous vaginal delivery

Early consultation with anesthesiology is recommended

Nursing:

Patients with chronic spinal cord injury require early medical intervention in labour and early obstetrical and anesthesia assessment due to the risk of autonomic dysreflexia

Analysis phase: allows learners to practice reflective learning by exploring and analyzing positive performances and performance gaps

Tips: 

- Be genuinely curious!

- Maintain the basic assumption that everyone is smart and is doing their best for the patient!

- Focus on two to three points including both technical and non-technical skills in your discussion

- Include discussion points based on your own observation as well as those generated by the learners in the ‘Reactions’ and “Descriptive’ phases

- Select appropriate tools including advocacy-inquiry, plus/delta, and directive feedback according to the type of performance gaps observed, the time available, the experience and insight of the learners, your experience as a debriefer.

“What went well? What would you have wanted to change or do differently?”

“What are some pros and cons of ... [observed action or behaviour]?”

 “What was going through your mind when you did/said ... ?”

 “What was your differential diagnosis?”

Normalize mistakes

“We specifically put you into a challenging situation and we did not expect you to manage everything perfectly”

“In our normal practice we are used to those around us acting in a specific way, things are different in the simulator”

Generalize to clinical practice

“Have you ever experienced anything similar in your practice?”

“What strategies have you seen people use in your clinical experience?”

Before moving to the ‘Summary’ phase ask the learners if there are any other issues they would like to discuss

Summary phase: allows learners to review and summarize what was learnt and to apply this to their clinical practice, and allows instructors to confirm if the learning objectives of the scenario were achieved

Tips:

- Avoid bringing up new discussion points or topics

- Ask the learners to summarize their main take-home points, i.e. “Tell me one thing you did well, one thing you would do differently, and one thing you learnt that you will apply to your clinical practice”

- Summarize the main points of the discussion

- Ensure everyone contributes, even learners who participated as confederates can share what they learnt from participating in the scenario and debriefing.

Appendix D: Evaluation form

Multidisciplinary Simulation

Date: _____________________________  Discipline: ____________________________         

Evaluation of the Session:

1 = strongly disagree, 2 = disagree, 3 = neutral, 4 = agree and 5 = strongly agree

1) The objectives of the session were explicitly stated                             1           2           3            4           5           N/A

2) The objectives were met by the simulation staff                                   1           2           3            4           5           N/A

3) Staff were knowledgeable and informed                                               1           2           3            4           5           N/A

4) There was sufficient time dedicated to hands-on                                 1           2           3            4           5           N/A

      practice

5) During the debriefing the staff provided appropriate                           1           2           3            4           5           N/A

      and timely feedback

6) The debriefing session was organized and helped                             1           2           3            4           5           N/A

      clarify important issues with the case

7) The knowledge gained from this session will influence                      1           2           3            4           5           N/A

      my practice

8) I have gained increased confidence in my capacities                         1           2           3            4           5           N/A

        to provide care to patients in acute emergencies

9) I would enjoy having more opportunities for practice                         1           2           3            4           5           N/A

Evaluation of the Debriefers:

1 = strongly disagree, 2 = disagree, 3 = neutral, 4 = agree and 5 = strongly agree

Name of Debriefers: ________________________________________                                                  

1) The debriefers provided a safe environment to share and              1           2           3            4           5           N/A

        discuss

2) The debriefers demonstrated professionalism throughout             1           2           3            4           5           N/A

         the debriefing

3) The debriefers were engaging and facilitated my learning             1           2           3            4           5           N/A

Evaluation of the Scenario:

1 = strongly disagree, 2 = disagree, 3 = neutral, 4 = agree and 5 = strongly agree

1) The scenario was relevant to my practice                                                  1           2           3            4           5           N/A

2) The clinical encounter had high realism                                                    1           2           3            4           5           N/A

3) The degree of difficulty of the scenario was appropriate                        1           2           3            4           5           N/A

      for my level of expertise

4) The case construct was conceptually sound                                            1           2           3            4           5           N/A

      (i.e., it made sense…)

If you have answered ≤ 2 to any of the previous statements, please briefly justify:

Global Evaluation of the experience:

1 = strongly disagree, 2 = disagree, 3 = neutral, 4 = agree and 5 = strongly agree

1) This was an effective use of my time                                                     1           2           3            4           5           N/A

2) I would appreciate having more simulation sessions                          1           2           3            4           5           N/A

3) I have gained knowledge through this experience                             1           2           3            4           5           N/A

4) What was the most useful or important lesson you learned during this session?

5) Can you identify any component of this session that would need to be improved in order to make the learning experience even better?

6) Please indicate which CanMeds roles were addressed during today’s session (check all that apply)

☐ Medical Expert

☐ Collaborator

☐ Professional

☐ Health Advocate

☐ Communicator

☐ Manager

☐ Scholar

Appendix E: Suggested 4-year curriculum

**Table 5:** Appendix E: Ob/Gyn Anesthesiology Nursing Simulation Curriculum

YEAR 1	Autonomic Dysreflexia	Twin Breech Delivery
YEAR 2	MgSO4 Toxicity	Difficult Airway, Emergent Delivery
YEAR 3	Thyroid Storm	Amniotic Fluid Embolism
YEAR 4	Cord Prolapse with Abnormal Fetal Heart Rate	Post Partum Hemorrhage
